# Inhibition of malaria and babesiosis parasites by putative red blood cell targeting small molecules

**DOI:** 10.3389/fcimb.2024.1304839

**Published:** 2024-03-20

**Authors:** Patrice V. Groomes, Aditya S. Paul, Manoj T. Duraisingh

**Affiliations:** Department of Immunology & Infectious Diseases, Harvard T. H. Chan School of Public Health, Boston, MA, United States

**Keywords:** malaria, babesiosis, host-directed inhibitors, *plasmodium*, *babesia*, erythrocyte, eryptosis, phosphatidylserine

## Abstract

**Background:**

Chemotherapies for malaria and babesiosis frequently succumb to the emergence of pathogen-related drug-resistance. Host-targeted therapies are thought to be less susceptible to resistance but are seldom considered for treatment of these diseases.

**Methods:**

Our overall objective was to systematically assess small molecules for host cell-targeting activity to restrict proliferation of intracellular parasites. We carried out a literature survey to identify small molecules annotated for host factors implicated in *Plasmodium falciparum* infection. Alongside *P. falciparum*, we implemented *in vitro* parasite susceptibility assays also in the zoonotic parasite *Plasmodium knowlesi* and the veterinary parasite *Babesia divergens*. We additionally carried out assays to test directly for action on RBCs apart from the parasites. To distinguish specific host-targeting antiparasitic activity from erythrotoxicity, we measured phosphatidylserine exposure and hemolysis stimulated by small molecules in uninfected RBCs.

**Results:**

We identified diverse RBC target-annotated inhibitors with *Plasmodium*-specific, *Babesia-*specific, and broad-spectrum antiparasitic activity. The anticancer MEK-targeting drug trametinib is shown here to act with submicromolar activity to block proliferation of *Plasmodium* spp. in RBCs. Some inhibitors exhibit antimalarial activity with transient exposure to RBCs prior to infection with parasites, providing evidence for host-targeting activity distinct from direct inhibition of the parasite.

**Conclusions:**

We report here characterization of small molecules for antiproliferative and host cell-targeting activity for malaria and babesiosis parasites. This resource is relevant for assessment of physiological RBC-parasite interactions and may inform drug development and repurposing efforts.

## Introduction

**Figure 1:**
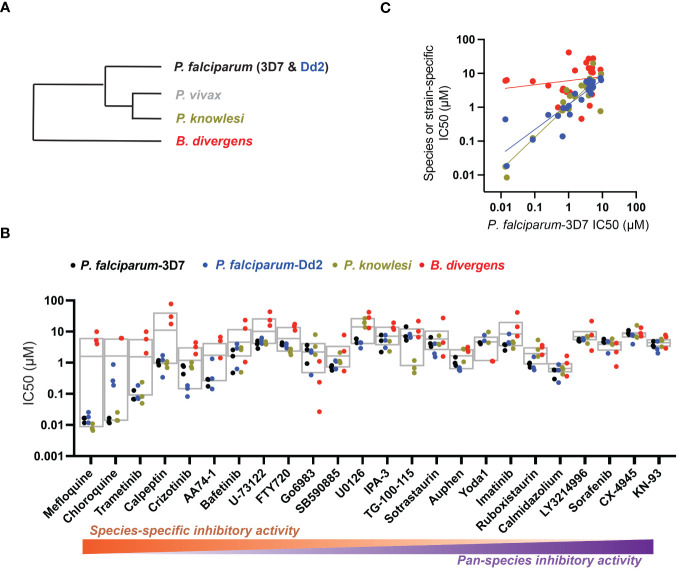
Host-biased inhibitors impede parasite intraerythrocytic proliferation. **(A)** Phylogeny of RBC-specific apicomplexan parasites in this study. **(B)** IC50 values in individual dose-response experiments, with parasite species (or strain) indicated. Experimental inhibitors are sorted according to species-specific score as described in the Methods. Box plots indicate mean and range over species. **(C)** For each inhibitor, IC50 measured in each strain or species as in **(B)** plotted against reference strain *P. falciparum* 3D7. Colors as in B; correlation by linear regression analysis.

Drugs to treat parasitic infections are few due to both limited drug development as well as the frequent emergence of resistance. Acting against targets in *Plasmodium* spp. parasites, drugs developed to treat malaria in the modern era have been undermined by resistance (McClure and Day, 2014), with the current first-line antimalarial artemisinin showing signs of decreased clinical efficacy in endemic areas (Menard and Dondorp, 2017; Wicht et al., 2020). Babesiosis, caused by infection with *Babesia* spp. parasites, distantly related to *Plasmodium*, has fewer chemotherapeutic options. Babesiosis is frequently treated with atovaquone, a drug compromised by frequent emergence of resistance; or clindamycin-quinine, a drug combination with limited efficacy (Waked and Krause, 2022). Cousins in the apicomplexan phylum (**Figure 1A**), *Plasmodium* and *Babesia* spp. parasites infect host red blood cells (RBCs) to cause disease. Leveraging existing collections of antimalarial inhibitors, we and others demonstrated that *Babesia* spp. parasites are susceptible to many inhibitors that also block *Plasmodium* spp., suggesting common processes for infection targeted by these inhibitors (Paul et al., 2016; Voorhis et al., 2016; Rizk et al., 2019; Rizk et al., 2021).

Host-directed therapies (HDTs) are used successfully to treat bacterial and viral infections and are thought to be more resilient to emergence of resistance than drugs targeting pathogen factors (Kaufmann et al., 2018). For *Plasmodium* and *Babesia* spp. that engage in intimate interactions with host cells virtually the entire parasite life cycle, delineation of parasite- versus host-specific activity for antiproliferative small molecules is a major challenge. Indeed, chemical inhibitors directed at factors with homologs across eukaryotes may directly interfere with both parasite and host targets. Despite the potential (Prudencio and Mota, 2013; Arang et al., 2017; Wei et al., 2021), there are few systematic descriptions for criteria and methods to establish host-directed activity, particularly for the blood-stage where fewer experimental tools for assessing the RBC are available. Nonetheless, many host factors required for *Plasmodium* infection are already targets for drugs approved by the FDA, and some host-directed drugs are being explored for repurposing including the anticancer tyrosine kinase inhibitor Imatinib (Gleevec) currently in Phase II clinical trials (Chien et al., 2021).

Here, we sought to provide evidence for host-targeting chemical activity from a curated panel of compounds. From the literature we identified 23 RBC factors implicated or established in *P. falciparum* infection, and we selected for analysis 28 small molecules annotated to target these RBC factors. We applied a cross-species comparative analysis to small molecules by assessing antiparasitic activity in *Plasmodium* and *Babesia* spp. We further measured RBC-targeted activity in relation to restriction of parasite proliferation, as well as potential for erythrotoxicity for the compounds.

## Methods

### Compounds

Chloroquine was purchased from Sigma-Aldrich (Cat. No C6628), and mefloquine from Sigma-Aldrich (Cat. No. M2319) or USP (Cat. No. 1379059). Background information on putative host targets and sourcing of other experimental compounds are detailed in **Table 1**.

**Table 1:** Compounds used in this study and antiparasitic potency.

Compound (Molecular weight; logP)	Putative Host Target	Target Class	Probe/Drug/Clinical Development status (ClinicalTrials.gov identifier)	Inclusion Criteria (code)	References (Pubmed identification numbers)	Source	IC50 (S.D.), µM. n= 3 experiments (or 2 where indicated with *)			
							*P. falciparum*-3D7	*P. falciparum*-Dd2	*P. knowlesi*	*B. divergens*
*Auphen 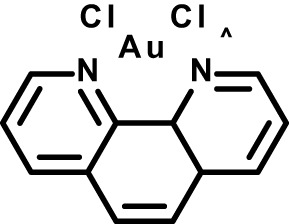 (483.5; n.a.)*	*AQP3*	*Channel*	*Probe*	*HF; Cpd (a,f)*	*29775485, 32330444*,	*Gift (E. Derbyshire, Duke Univ)*	*1.1 (0.4)*	*0.61 (0.07)*	*2 (1.0)*	*2.8 (0.5)*
*Yoda1 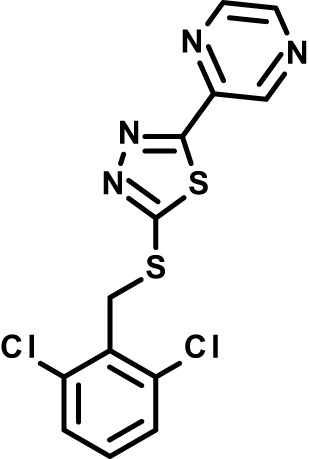 (355.3; 3.5)*	*PIEZO1*	*Channel*	*Probe*	*HF; Cpd (a,b,d)*	*26830761, 32265284, 29576450, 37071200*	*Tocris, Cat. No. 5586*	4.2 (0.3)*	6 (1.9)*	7 (4)*	1.11 (0.02)*
*Aurintricarboxylic acid (ATA)* 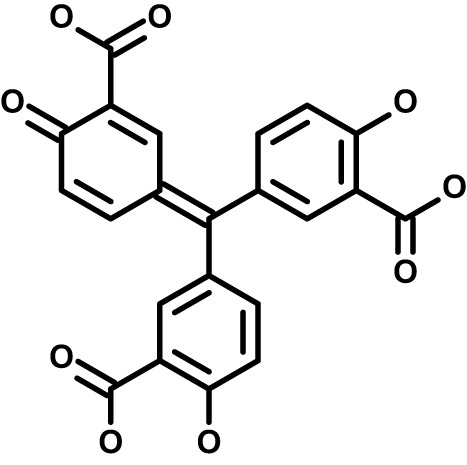 *(422.3; 4.3)*	*PMCA4*	*Channel*	*Probe*	*HF; Cpd (a,b,d)*	*25261933, 34215257, 35768835*	*Sigma-Aldrich, Cat. No. 189400*	*37 (7)*	*44 (8)*	*n.d.*	*29 (5)*
*BI 749327* 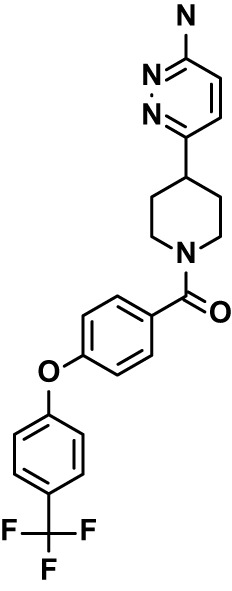 *(442.4; 3.6)*	*TRPC6*	*Channel*	*Probe*	*HF (d,e)*	*23332153*	*MedChemExpress, Cat. No. HY-111925*	*25 (2)*	*22 (4)*	*40 (15)*	*30 (15)*
*FTY720 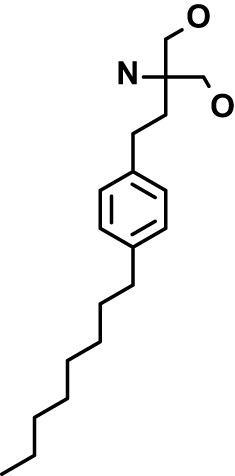 (343.9; 5.5^1^)*	*TRPM7*	*Channel; RBC Cytoskeleton*	*Drug*	*HF; Cpd (a,c,d,e)*	*28226242*	*Sigma-Aldrich, Cat. No. SML0700*	*3.9 (0.4)*	*3.5 (0.6)*	*2.2 (0.5)*	*14 (3)*
*TG-100-115 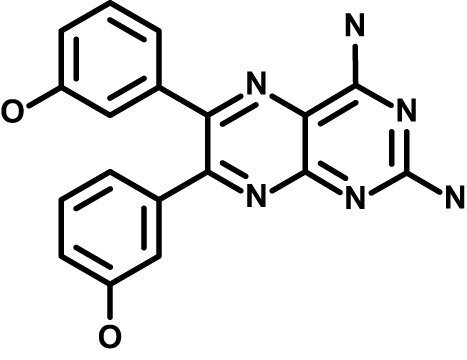 (346.3; 1.7)*	*TRPM7*	*Kinase domain; RBC cytoskeleton*	*Probe*	*HF (d,e)*	*28226242*	*Enzo Life Sciences, Cat. No. ENZ-CHM139-0005*	*9 (5)*	7 (1.4)*	*0.8 (0.4)*	*13 (8)*
*Imatinib mesylate 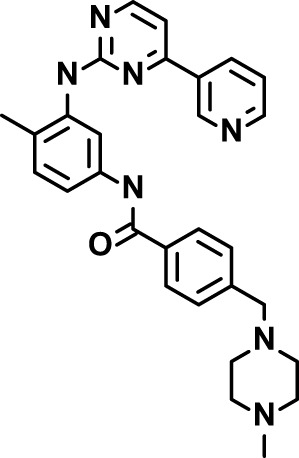 (493.6; 3.5)*	*SYK, LYN*	*Tyr Protein Kinase*	*Drug*	*HF; Cpd (a,d,e)*	*27768734, 28634183, 20799346*	*Enzo Life Sciences, Cat. No. ALX-270*	*3.3 (0.8)*	*5.7 (2.3)*	*4 (1.0)*	20 (17)*
*Bafetinib 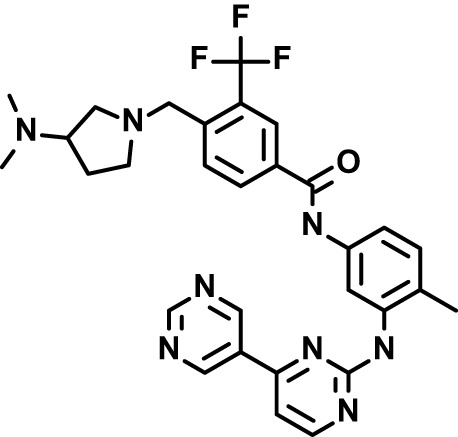 (576.6; 4.3)*	*LYN*	*Tyr Protein Kinase*	*Phase II (Suspended): NCT01144260*	*HF; Cpd. (a,d,e)*	*27768734, 28634183, 20799346*	*Selleck Chemicals, Cat. No. S1369*	*2 (1.0)*	*3 (2)*	*2 (1)*	*12 (11)*
*CX-4945 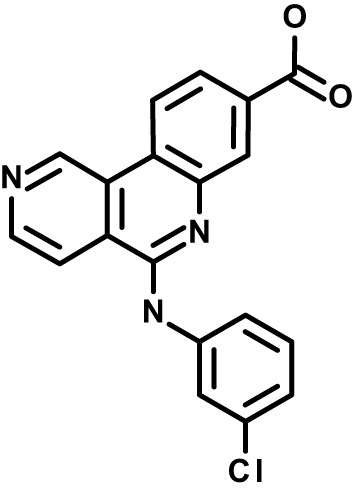 (349.8; 4.4)*	*CK2*	*Ser-Thr Protein Kinase*	*Phase II: NCT04663737*	*HF (d,e); footnote 3*	*26830761, 20861458, 19131328*	*Selleck Chemicals, Cat. No. S2248*	*9 (1.5)*	*6 (2)*	*9 (5)*	*10 (3)*
*Tetrabromocinnamic acid 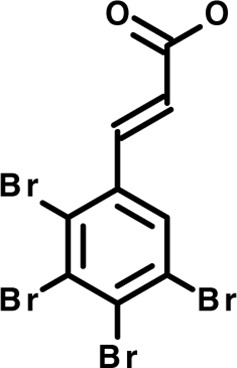 (463.7; 4.6)*	*CK2*	*Ser-Thr Protein Kinase*	*Probe*	*HF; Cpd (c,d,e)*	*26830761, 20861458, 19131328*	*Sigma-Aldrich, Cat. No. SML0854*	>100*	>85*	>80*	>80*
*Sotrastaurin 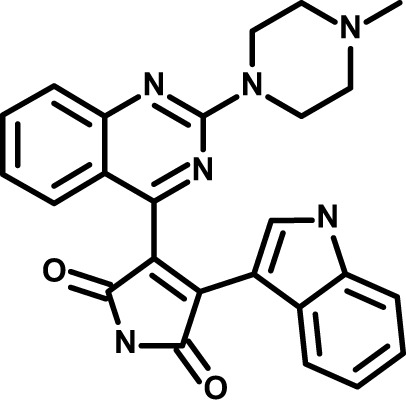 (438.5; 2.6)*	*PKC*	*Ser-Thr Protein Kinase*	*Phase II (Completed then suspended): NCT00885196*	*HF; Cpd (a,d,e)*	*23332153*	*Selleck Chemicals, Cat. No. S2791*	*5 (2)*	*3 (1)*	*5 (2)*	*10 (14)*
*Ruboxistaurin 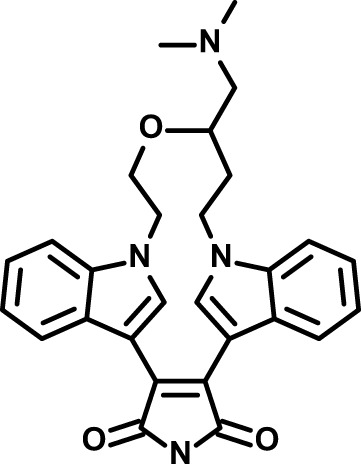 (468.5; 2.7)*	*PKC*	*Ser-Thr Protein Kinase*	*Phase III (Completed then suspended): NCT00090519*	*HF (d,e)*	*23332153*	*Selleck Chemicals, Cat. No. S7663*	*0.9 (0.14)*	*0.9 (0.5)*	*3 (2)*	*2.8 (0.9)*
*Go6983 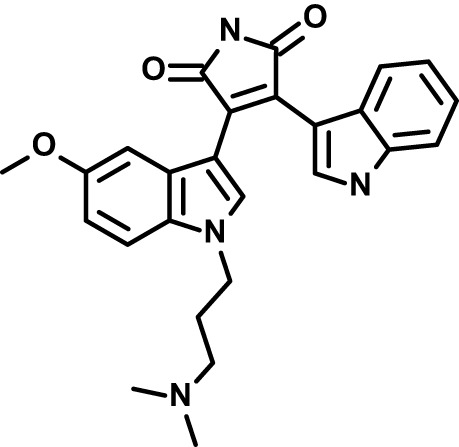 (442.5; 3.1)*	*PKC*	*Ser-Thr Protein Kinase*	*Probe*	*HF; Cpd (d,e)*	*23332153*	*Selleck Chemicals, Cat. No. S2911*	*2 (1)*	*2 (1)*	*4 (3)*	*0.5 (0.6)*
*KN-93 Phosphate 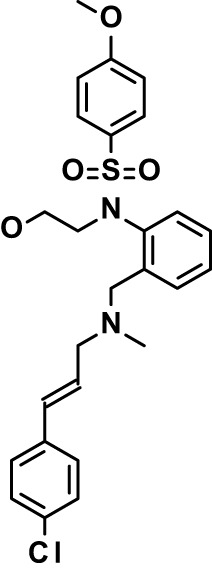 (599.03; n.a.)*	*CAMK2*	*Ser-Thr Protein Kinase*	*Probe*	*HF; Cpd (a,e)*	*26830761, 23332153, 20037583*	*Selleck Chemicals, Cat. No. S7423*	*3.9 (0.9)*	*3 (2)*	*5 (1.3)*	*6 (3)*
*Crizotinib 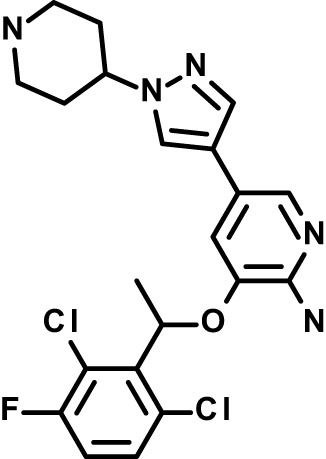 (450.3; 1.83^2^)*	*c-Met*	*Tyr Protein Kinase (MAPK)*	*Drug*	*HF; Cpd (a,d)*	*32782246*	*Selleck Chemicals, Cat. No. S1068*	*0.7 (0.2)*	*0.14 (0.05)*	*0.8 (0.2)*	*3 (1.3)*
*SB590885 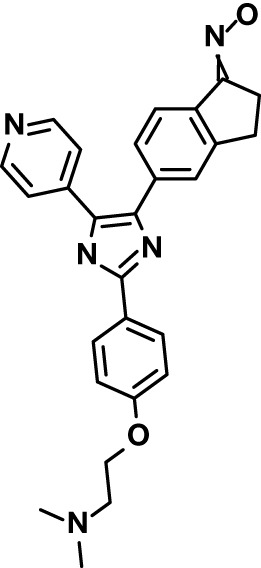 (453.5; 4)*	*B-Raf*	*Ser-Thr- Protein Kinase (MAPK)*	*Probe*	*HF; Cpd (a,d)*	*32782246*	*Selleck Chemicals, Cat. No. S2220*	*0.7 (0.12)*	*1.0 (0.3)*	*1.4 (0.6)*	*4 (4)*
*Sorafenib 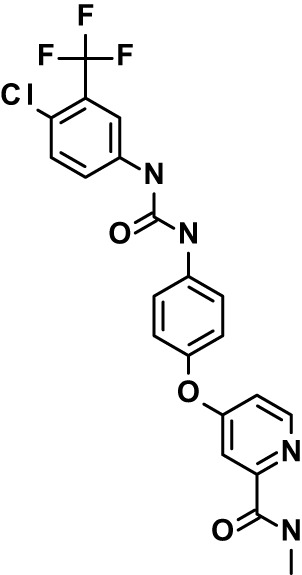 (464.8; 4.1)*	*B-Raf, C-Raf*	*Ser-Thr- Protein Kinase (MAPK)*	*Drug*	*HF; Cpd (a,d)*	*32782246*	*Selleck Chemicals, Cat. No. S7397*	*4.9 (0.7)*	*4 (2)*	*5 (1.1)*	*2 (2)*
*GW5074 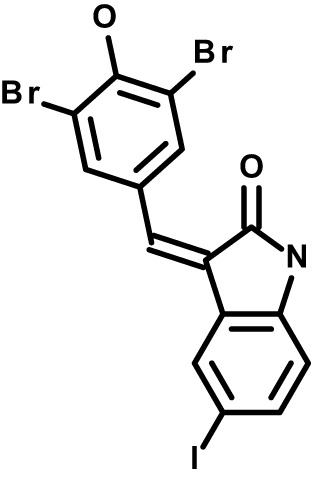 (520.94; 4.6)*	*C-Raf*	*Ser-Thr- Protein Kinase (MAPK)*	*Probe*	*HF; Cpd (a,d)*	*32782246, 36430384*	*Selleck Chemicals, Cat. No. S2872*	*19 (6)*	*13 (3)*	10 (7)*	*30 (13)*
*Trametinib 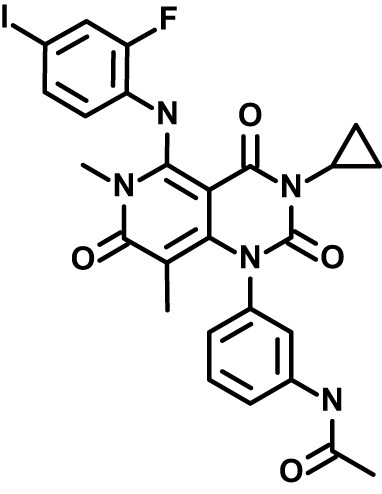 (615.4; 3.4)*	*MEK1*	*Ser-Thr-Tyr Protein Kinase (MAPK)*	*Drug*	*HF; Cpd (a,c,d)*	*32782246, 28679535, 21371233*	*Selleck Chemicals, Cat. No. S2673*	*0.09 (0.03)*	*0.11 (0.07)*	*0.1 (0.10)*	*6 (4)*
*U0126 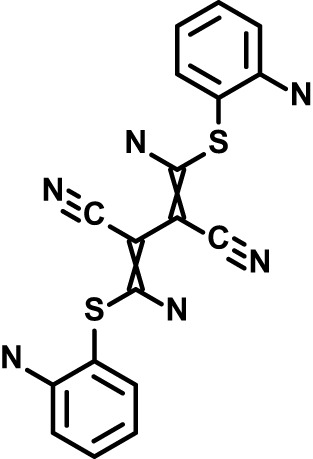 (380.5; 2)*	*MEK1*	*Ser-Thr-Tyr Protein Kinase (MAPK)*	*Probe*	*HF; Cpd (a,d)*	*32782246, 28679535, 21371233*	*Sigma-Aldrich, Cat. No. 19-147*	*5.3 (0.9)*	*3.9 (0.9)*	*20. (6)*	*30 (15)*
*IPA-3* 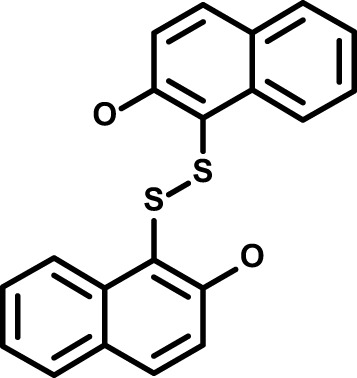 *(350.5; 5.8)*	*PAK1*	*Ser-Thr Protein Kinase (MAPK)*	*Probe*	*HF; Cpd (a,d)*	*32782246, 28679535, 21371233*	*Selleck Chemicals, Cat. No. S7093*	*5 (3)*	*5 (2)*	4 (1.8)*	*15 (4)*
*LY3214996 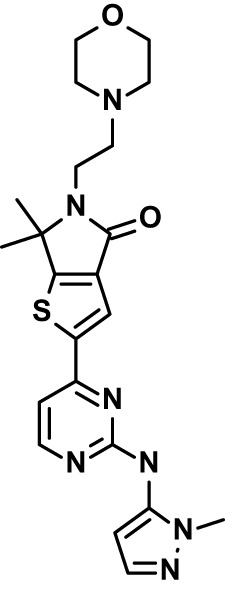 (453.6; 1.4)*	*ERK1 and ERK2*	*Ser-Thr Protein Kinase (MAPK)*	*Phase I: NCT04033341*	*HF (d)*	*32782246, 28679535, 21371233*	*Selleck Chemicals, Cat. No. S8534*	*5.2 (0.5)*	*5.7 (0.3)*	*6 (2)*	*10 (10)*
*AZD0364 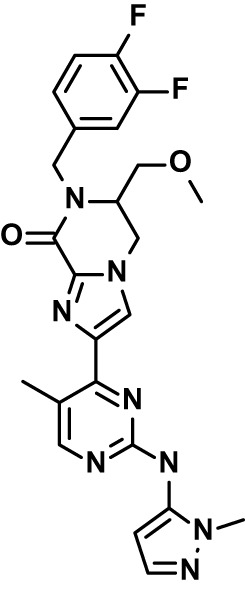 (494.5; 2)*	*ERK2*	*Ser-Thr Protein Kinase (MAPK)*	*Probe*	*HF (c,d)*	*32782246, 28679535, 21371233*	*Selleck Chemicals, Cat. No. S8708*	*16 (1.9)*	*13 (3)*	*n.d.*	*16 (5)*
*AA74-1 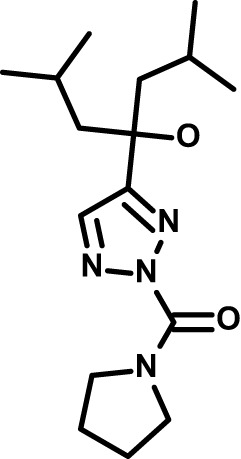 (308.4; 3)*	*APEH*	*Peptidase, Deacetylase*	*Probe*	*Cpd (a)*	*31068431*	*Sigma-Aldrich, Cat. No. SML0358*	*0.25 (0.07)*	*0.5 (0.6)*	*n.d.*	*4 (3)*
*Calmidazolium 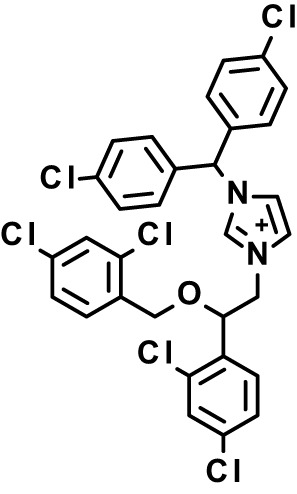 (652.2; 10)*	*Calmodulin*	*Regulatory*	*Probe*	*HF; Cpd (a,e)*	*23332153, 3541784*	*Sigma-Aldrich, Cat. No. 208665*	*0.5 (0.2)*	*0.6 (0.3)*	*0.6 (0.2)*	*1.2 (0.6)*
*Calpeptin 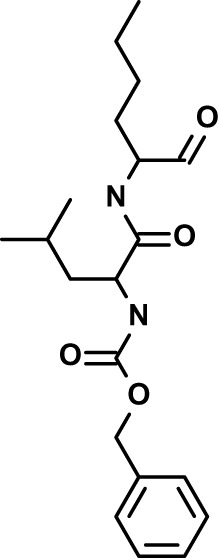 (362.5; 3.9)*	*Calpain-1*	*Protease*	*Probe*	*HF (e); footnote 4*	*23332153;* 19342550	*Selleck Chemicals, Cat. No. S7396*	*1.0 (0.2)*	*1.3 (0.7)*	*0.9 (0.2)*	*40 (30)*
*U-73122 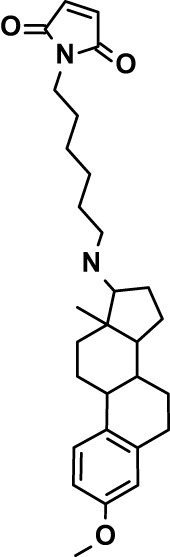 (464.6; 4)*	*PLC*	*Phospholipase*	*Probe*	*HF (e); Cpd (a)*	*23332153; 34819379*	*Sigma-Aldrich, Cat. No. U6756*	*4 (1.1)*	*5 (1.1)*	*4.4 (0.5)*	*20 (14)*
*MBQ-167 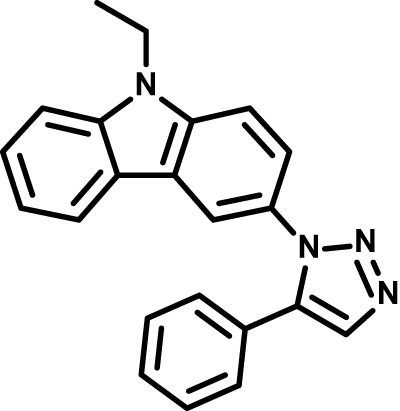 (338.4; 4.7)*	*Rac1 and Rac2*	*GTPase*	*Probe*	*HF (c,d)*	*16882712, 33328606*	*Selleck Chemicals, Cat. No. S8749*	*33 (3)*	*23 (3)*	*38 (4)*	40. (6)*

Small molecules were selected on the basis of evidence for antimalarial activity by compound (Cpd) or implication of targeted host factor in *Plasmodium* function (HF), with codes below providing further details:

a. Established antimalarial inhibitor.

b. Under selection in malaria-endemic regions.

c. RBC phenotype elicited by compound or mutant.

d. Host pathway experimentally associated with *P. falciparum* infection in RBCs.

e. Host factor implicated in apicomplexan invasion or egress.

f. Activity against liver stage infection.

Octanol-water partition coefficients (logP) are predicted values obtained from the PubChem database (https://pubchem.ncbi.nlm.nih.gov/) unless indicated otherwise with superscript annotation:

1. FTY720, experimental logP. https://go.drugbank.com/drugs/DB08868.

2. Crizotonib, experimental logP. https://go.drugbank.com/drugs/DB08865.

Miscellaneous information for specific compounds:

3. CX-4945, nanomolar inhibition demonstrated against recombinant *P. falciparum* Casein Kinase 2 in biochemical assay, PMID 29743645.

4. Calpeptin, >100µM application of inhibitor blocks native turnover of *P. falciparum* antigen AB4 in live parasites, PMID 26893139.

### Parasite culture

The *P. falciparum* strains 3D7 and Dd2 were obtained from the Walter and Eliza Hall Institute (Melbourne, Australia) and the Malaria Research and Reference Reagent Resource, respectively. The *P. knowlesi* YH1 strain adapted for growth in human erythrocytes and the BdC9 line of *B. divergens* were previously reported (Lim et al., 2013; Rezvani et al., 2022). All parasite lines were maintained in continuous culture in human erythrocytes O+ purchased from a commercial source (Research Blood Components, Boston USA) in RPMI-1640 medium as described (eg Rezvani et al., 2022), typically at 2%-hematocrit. *Plasmodium* parasites were synchronized using sorbitol (5% [wt/vol] for both *P. falciparum* and *P. knowlesi*; for *P. knowlesi*, with addition of 20 mM Na-HEPES and 0.1 mg/ml BSA) (Lambros and Vanderberg, 1979) at least once in the week prior to assays. *B. divergens* parasites were maintained and assayed in mixed stages without synchronization.

### Inhibitor susceptibility assays

We carried out dose-response assays in 384-well plates (40 µl assay volume) for measurement with SYBR-Green I dye (Mandt et al., 2019), with test compounds pre-printed into wells via use of a D300 Digital Dispenser (Hewlett-Packard). Ring-stage *P. falciparum* (1%-parasitemia starting, 1%-hematocrit) and asynchronous *B.divergens* (0.5%-parasitemia starting, 3%-hematocrit) were incubated with plated compounds for 72h, and ring-stage *P. knowlesi* (1%-parasitemia starting, 1%-hematocrit) for 48h, before lysis and staining with dye. To quantify the species-specificity of the inhibitors, we divided the range of IC50s across the 4 test lines by the average IC50 across the same lines: inhibitors with increased scores show species-specific activity and reduced scores indicate broad-spectrum, pan-species activity.

### Assay for inhibition of parasite proliferation via RBC-targeting

We printed test compounds onto duplicate U-bottom 96-well plates (5 technical replicates at single dose; 5x *P. falciparum* 3D7-IC50 for 100µl final volume, 1% maximum DMSO v/v). All plates were frozen for storage before assay. For assay, duplicate plates were preincubated with donor O+ RBCs at 2% hematocrit at 37°C in 50µL for 4-5 hours. Following preincubation, one but not the other plate in each pair was washed 4X in complete RPMI to dilute inhibitor. We transferred RBCs from both washed and unwashed plates to black, clear bottom 96-well plates (Corning Cat. No. 3904) and supplemented these suspensions with MACS-purified *P. falciparum* (3D7) schizonts for a starting parasitemia of 0.1% - 0.25% at 1% hematocrit. Following 72h incubation in standard culture conditions, samples were processed for SYBR-Green I signal (Mandt et al., 2019). Following normalization of SYBR-signal to DMSO vehicle (0.01%) and 10 µM staurosporine (Selleck Chemical, cat. no. S1421), a 1000x *Pf*3D7-IC50 dose that remains inhibitory after dilution, we quantified for each compound retained inhibitory activity following washing of RBCs [100%-(%SYBR_washed_ – %SYBR_unwashed_)]. To identify host-targeting compounds, we compared DMSO-treated to inhibitor-treated samples (Students t-test, paired). We carried out the assay with 1 µM mefloquine (Sigma-Aldrich Cat. No. M2319) as a control for reversible inhibition. All assays were conducted in biological triplicate, with each experiment utilizing different donor RBCs. For select compounds, we carried out this same assay in a dose-response series (n=3 experiments). Following normalization to DMSO-controls, we assessed inhibitor activity following transient exposure of RBCs by Two-Way ANOVA tests for repeated measures (Graphpad Prism v9): high *P* values indicate support for the null hypothesis that dose-dependent inhibition is not modulated by dilution after exposure, consistent with a direct inhibitory effect of the compounds on RBCs persisting after transient exposure.

### Cellular phosphatidylserine exposure and hemolysis assays

We used a MACSQuant Analyzer flow-cytometer (Miltenyi Biotec) to assess surface-exposed phosphatidylserine on RBCs with fluorescent annexin V (Kuypers et al., 1996). We added 100 µl samples of RBCs (1%-hematocrit) in RPMI, to pre-printed compounds in 96-well format (5x IC50, technical duplicate). At 24, 48, and 72h, we resuspended RBCs, diluted and washed 5 µl samples in Annexin V Buffer (2.5mM Calcium Chloride; 140mM Sodium Chloride; 10mM HEPES; pH 7.4), and stained with Annexin V-488 (Invitrogen Cat. No. A13201) diluted 1:100 in Annexin V Buffer for 15min in the dark before measurement (Jezewski et al., 2021). We analyzed samples using a volumetric MACSQuant Analyzer Flow Cytometer (Miltenyi Biotec), manually resuspending each row immediately prior to sample uptake. We recorded 10,000 total events for each condition at the instrument-defined low flow rate. We assessed all compounds in 2 separate experiments with blood from different donors. Based on gates derived from samples treated with calcium ionophore A23187 (10 µM) as a standard for Annexin V-positive signal at each timepoint (Wesseling et al., 2016), we determined in each sample the percentage of RBCs that displayed phosphatidylserine; we calculated hemolysis based on the cell count per unit of time. All samples were gated for single cell events.

## Results

To assess host cell-targeting as a strategy to restrict malaria and babesiosis parasites, we carried out a literature survey for RBC targets implicated in malaria infections and small molecules that target these factors. We selected putative targets on the basis of reports of involvement in *P. falciparum* infection, host genetic or chemical biological evidence for apicomplexan parasite infection, and signatures of selection from population genetics (**Table 1**). Of 28 compounds annotated for 23 host targets, 20 compounds are previously reported to restrict *P. falciparum* proliferation.

Related to chemotherapeutic potential, 10 of the small molecules are additionally FDA-approved drugs or have a track record of clinical development (**Table 1**). To evaluate the cross-parasite species antiparasitic activity of host-biased inhibitors, we implemented chemical susceptibility assays (**Figures 1B, C**) with: 1) *P. falciparum* strain 3D7, a chloroquine-sensitive strain; 2) *P. falciparum* strain Dd2, a chloroquine-resistant strain; 3) a human adapted strain YH1 of the simian malaria parasite *P. knowlesi*, a zoonotic pathogen that frequently serves as a surrogate in the lab for culture-refractory *Plasmodium vivax* (Grüring et al., 2014); and 4) strain BdC9 of *Babesia divergens*, a zoonotic bovine pathogen that grows readily *in vitro* in human RBCs (Lobo et al., 2019). Alongside the well-established antimalarials chloroquine and mefloquine, we observed in the reference species *P. falciparum* 22 compounds with IC50<10 µM (**Table 1**), which we considered for further analysis.

Our experimental compound set is weighted toward kinase inhibitors, reflecting the high degree of small molecule development in this target space. The MAPK pathway-targeting drugs trametinib and crizotinib were previously reported to exhibit antimalarial activity *in vivo* and *in vitro*, respectively (Wu et al., 2017; Adderley et al., 2020), and we observed here potent submicromolar activity *in vitro* for the *Plasmodium* spp. parasites and single-digit micromolar activity in *B. divergens* (**Figure 1B**). We additionally observed sub- to single-digit micromolar activity for other preclinical and clinical compounds including the CK2 inhibitor CX-4945 and the PKC inhibitor ruboxistaurin reported for the first time here to exhibit antiparasitic activity (**Figure 1B**). Compounds were highly correlated in their activities across the *Plasmodium* spp., in a manner independent of chloroquine-resistance in *P. falciparum* (**Figures 1B, C**), similar to patterns of cross-species correlation we and others have reported (Paul et al., 2016; van Schalkwyk et al., 2017). Interestingly, TG-100-115, an inhibitor for the kinase domain of the channel TRPM7, without previously documented antiparasitic activity, showed submicromolar activity against *P. knowlesi* ~10x stronger than exhibited against the other parasite species. Apart from the antimalarial drugs chloroquine and mefloquine, trametinib (IC50 ~0.1 µM) and calpeptin (IC50 ~1 µM) showed the highest specific activity against the *Plasmodium* spp. lines, with >30-fold higher doses required for 50% inhibition of *B. divergens*. For most compounds, we observed weaker activity against *B. divergens* than the *Plasmodium* spp. lines; Piezo1 activator Yoda1 and PKC inhibitor Go6983 are therefore notable for at least 3-fold lower IC50s against *B. divergens* than the *Plasmodium* spp. (**Figures 1B-C**; **Supplementary Figure 1**). We note that host PKC has been previously implicated in RBC infection by *P. falciparum* as well as non-RBC host cell infection by distantly related *Toxoplasma gondii* (Millholland et al., 2013; Adderley et al., 2020); our data showing activity for multiple PKC inhibitors in *B. divergens* is consistent with a broadly conserved role for host PKC in infection by parasites from Apicomplexa (**Figure 1**, **Table 1**). Our analysis also revealed compounds with similar micromolar potency in *Plasmodium* spp. and *B. divergens*, including MAPK inhibitors LY3214996 and Sorafenib, CX-4945, and CamK2 inhibitor KN-93. For some inhibitors for which we observed here antiparasitic activity not previously reported, we tested the potential of increased potency in *P. falciparum* in co-administration with established antimalarials. In combination with fixed, sublethal doses of the test compounds, we found modest (≤2-fold), statistically significant (*P*<0.05) reductions in the IC50 of chloroquine with ruboxistaurin, and both chloroquine and mefloquine with trametinib (**Supplementary Figure 2**).

**Figure 2:**
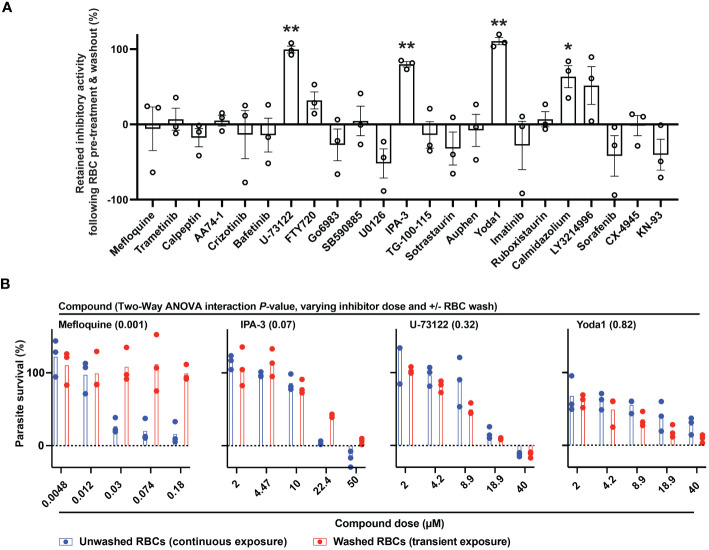
Identifying host-targeting antiparasitic compounds. **(A)** Retained inhibitory activity following transient exposure to RBCs preceding parasite infection. Dose, 5x IC50 for *P. falciparum*; n=3 experiments; 100% = inhibition with continuous exposure. Mean +/- s.e.m. Student’s t test; *P*: [*],<0.05; [**],<0.01. **(B)** Dose-response profiles of parasite infection and proliferation, for the indicated compounds, with either continuous or transient exposure of RBCs to inhibitor at the indicated micromolar doses. N=3 experiments; Two-Way ANOVA with Interaction *P-*value.

We considered that transient exposure of RBCs to inhibitors prior to parasite infection may provide a test for host-targeting activity distinguishable from direct targeting of the parasite. We measured proliferation of *P. falciparum* in RBCs that were pre-treated with a high dose of compound and washed extensively to dilute free inhibitor before exposure to parasites (Brizuela et al., 2014; Sisquella et al., 2017). With this assay, we identified small molecules that retained inhibitory activity comparable to conditions wherein RBCs are maintained in the presence of inhibitor (**Figure 2A**). By this standard, while most compounds lost activity upon dilution, MAPK pathway inhibitor IPA-3, PLC inhibitor U-73122, and Yoda1, each showed antimalarial activity at levels ≥80% observed with continuous exposure to inhibitor (*P*<0.01). For these selected compounds, we carried out secondary assays in a dose-responsive format. Control compound mefloquine does not retain antimalarial activity at any dose following transient exposure to RBCs (**Figure 2B**). IPA-3, U-73122, and Yoda1, by contrast, demonstrate durable inhibitor activity following transient exposure statistically indistinguishable from inhibition with continued exposure. Critically, at sublethal doses at which inhibitor activity is not saturating, potencies with transient exposure are comparable to continued exposure (**Figure 2B**), suggesting durable effects with negligible contribution of free inhibitor remaining after washing of RBCs. These results are consistent with host-targeting as a primary mode of restriction of parasite proliferation for IPA-3, U-73122, and Yoda1.

**Figure 3:**
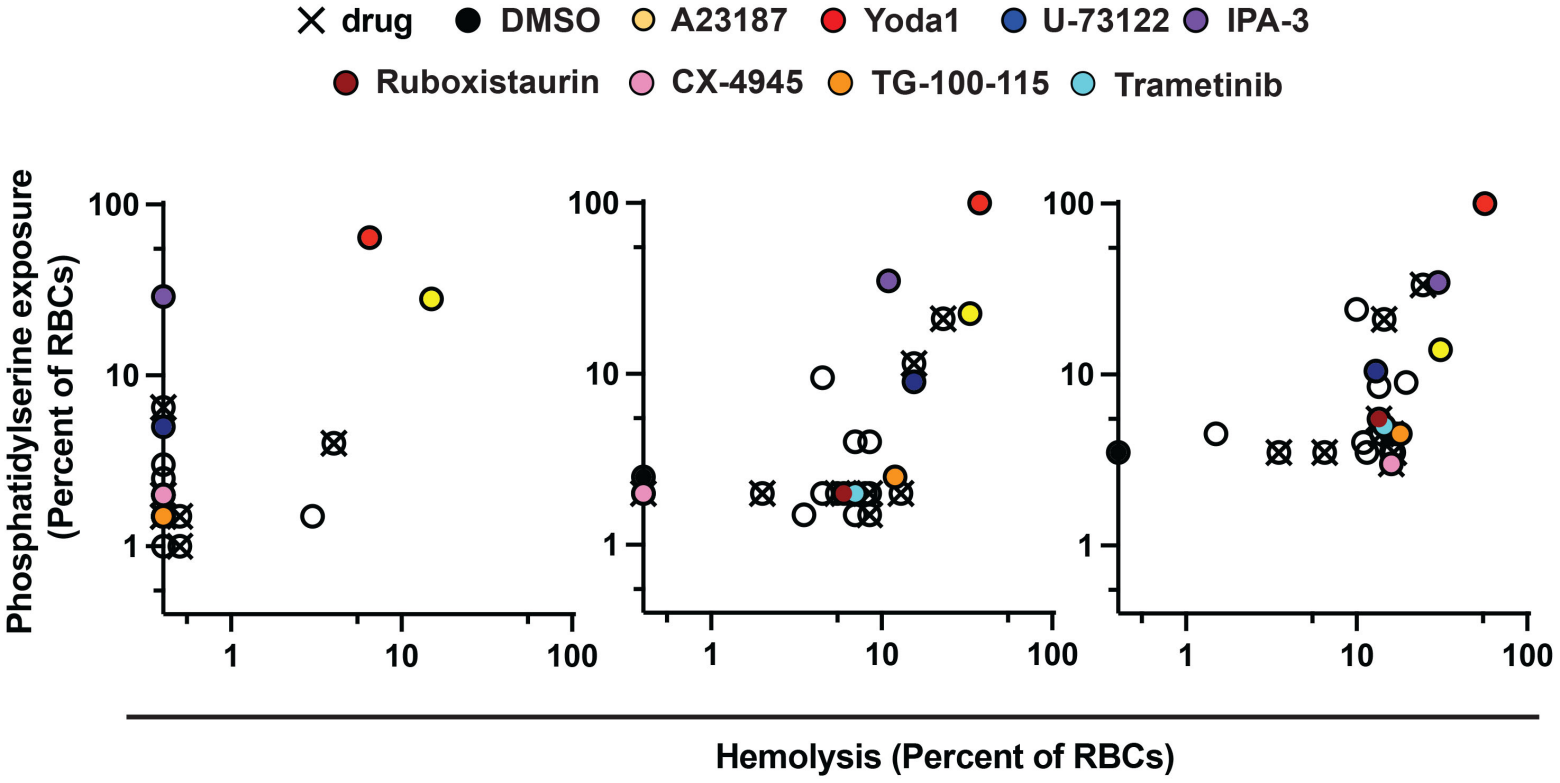
Erythrotoxicity of host-biased compounds. Phosphatidylserine (PS) exposure and hemolysis of RBCs following 24, 48, and 72h of compound treatment (5x *P. falciparum* 3D7-IC50); mean of 2 experiments. Each datapoint is related to a compound, with all source data reported in **Supplementary Table 1**. Labels for compounds of specific emphasis are indicated in the graphic.

RBC-targeting inhibitors may elicit toxicity through off-target activity or activity secondary to engagement of the primary, annotated target. Some drugs stimulate reactive oxygen species (Deavall et al., 2012), and for erythrocytes oxidative damage can induce eryptosis and rapid clearance from the bloodstream (Dreischer et al., 2022; Scovino et al., 2022). As a proxy for erythrotoxic action of small molecules, we measured phosphatidylserine (PS) exposure (Dreischer et al., 2022; Scovino et al., 2022) and hemolysis following treatment of RBCs by flow-cytometry. Among the durably acting antimalarial inhibitors (**Figure 2**), Yoda1 and IPA-3 elicited the most profound effect (~30-60% of RBCs with PS exposure at 24h), with Yoda1 also exhibiting a rapid onset of hemolysis comparable to control compound A23187 (**Figure 3**; **Supplementary Table 1**) – both compounds have previously been reported to stimulate eryptosis (Zelenak et al., 2011; Wadud et al., 2020). U-73122 stimulates lower levels of hemolysis and PS exposure on RBCs, within the range of some fully reversible inhibitors in the panel including approved drugs. Trametinib, ruboxistaurin, CX-4945, and TG-100-115, elicit similarly low levels of hemolysis and PS exposure (**Figure 3**; **Supplementary Table 1**).

## Discussion

The specific susceptibility of *Plasmodium* spp. to calpeptin is consistent with previous work showing the importance of host calpain-1 for *P. falciparum* egress from RBCs (Chandramohanadas et al., 2009; Millholland et al., 2013). Studies of egress in *Babesia* spp. have focused on parasite factors (Asada et al., 2012; Paoletta et al., 2021; Elsworth et al., 2023), and our work showing the relative insensitivity of *B. divergens* to calpeptin may well suggest differential utilization of host protease pathways compared to its malaria parasite cousin. By the same token, further work is required to understand the elevated sensitivity of *B. divergens* to the PIEZO1 channel activator Yoda1 compared to activity in *P. falciparum*. Our work suggests that *B. divergens* may be affected by factors like calcium influx and dehydration elicited by the compound and channel activity (Cahalan et al., 2015).

In *P. falciparum-*infected RBCs, chemical targeting of the host PAK-MEK axis of the MAPK pathway restricts parasite proliferation (Sicard et al., 2011). Notwithstanding weak activity in *B. divergens*, the strongest inhibitor in our experimental panel is the inhibitor of MEK and anticancer drug trametinib. In a mouse model of severe malaria with *Plasmodium berghei*, trametinib was shown to improve survival of infected mice (Wu et al., 2017). Though improvement of infection outcomes with trametinib may also be mediated through immune mechanisms (Wu et al., 2017), our *in vitro* work shows that the drug also acts directly on the parasite-erythrocyte complex to reduce proliferation (**Figures 1**–**3**).

Treatment with pro-eryptotic small molecules have been suggested as a malaria therapy (Boulet et al., 2018); our findings underscore that such an approach will likely also affect uninfected RBCs and would need careful evaluation. In terms of hemolysis and PS exposure, we observed diverse cellular responses to compound treatment. Previous work demonstrated that enolase inhibitors stimulate PS exposure on RBCs without hemolysis (Jezewski et al., 2021); our data over a panel of compounds with diverse targets suggest an association between the two parameters, with PS acting as a leading indicator for hemolysis (**Supplementary Figure 3**; **Supplementary Table 1**). Our study further provides evidence that erythrotoxic small molecules (**Figure 3**) have a propensity for inducing effects in RBCs to reduce parasite proliferation (**Figure 2**) that persist well after levels of free inhibitor decline. Durable antimalarial effects of inhibitors in RBCs may be related to stable ligation to a specific molecular target or targets by the compound, secondary effects in the host cell that persist after transient exposure to the compound, and/or sequestration of the compound by the RBC. The induction of erythrotoxicity by Yoda1 and IPA-3 suggests that effects downstream of primary action of the compounds on RBC ion homeostasis and signaling, respectively, render RBCs refractory to infection and proliferation. Indeed, a recent study reports increased intracellular calcium, increased fragility, and morphological changes in RBCs treated with Yoda1 (Lohia et al., 2023). The same study also reports minimal to weak antimalarial activity following transient exposure of RBCs to the compound; we may have elicited stronger effects owing to use of higher concentrations of Yoda1 and longer incubation with RBCs. Many bioactive compounds, including the small molecules in this study, are lipophilic (**Table 1**), and continued association with host cell plasma membranes or modification upon entry into the RBC to effectuate sequestration may facilitate antiparasitic potency during infection. Interestingly, in melanoma patients trametinib was observed to accumulate onto RBCs (Isberner et al., 2022); given our findings that an effect of the drug on the RBC does not persist beyond transient exposure, association of trametinib with the host cell is likely dynamic. We note that in our measurements across a broad panel of compounds, we observed minimal PS exposure for both FTY720 and Sorafenib (**Supplementary Table 2**), each of which have otherwise been reported in individual studies to stimulate the signal on erythrocytes (Eberhard et al., 2010; Lupescu et al., 2012).

Altogether, our studies provide a resource and framework to investigate the determinants of host targeting in RBC-specific apicomplexan parasites at greater resolution. We identified broad-spectrum and species-specific inhibitors, providing tools to understand shared as well as divergent host determinants of infection. We suggest that incorporation of analysis of erythrotoxicity is important for study of mechanism of action and may be useful to prioritize compounds in screening efforts oriented around translation. We found that many of the compounds exhibit IC50s for *P. falciparum* inhibition similar to reports for potency in cellular assays in uninfected RBCs and other mammalian cell types (**Supplementary Table 2**), and further investigation is required to distinguish parasite from host targeting for these inhibitors. There are few *in situ* methods to directly observe perturbation of specific targets in cells, and in general these approaches do not lend themselves to higher throughput analysis. Thermal proteome profiling (TPP) holds promise as an unbiased biochemical approach for assessing targets; as yet, use in parasites is limited (Dziekan et al., 2019; Lu et al., 2020; Hanna et al., 2023), and to our knowledge TPP has not been used to distinguish host- from parasite-targeting. In addition to biochemical approaches to assess direct engagement, we anticipate that the use of chemical genetic approaches in RBCs (eg Scully et al., 2019) will be powerful in establishing host targets in the context of parasite infection.

## Data availability statement

The original contributions presented in the study are included in the article and **Supplementary Material**. Further inquiries can be directed to the corresponding author.

## Ethics statement

This study was deemed not subject to human subjects research guidelines by the Harvard Longwood Campus Institutional Review Board, because the researchers do not have access to identifying information for donors of the commercially purchased human blood used in the investigation.

## Author contributions

PG: Investigation, Methodology, Conceptualization, Funding acquisition, Project administration, Writing – original draft. AP: Writing – original draft, Writing – review & editing, Formal analysis, Investigation, Visualization. MD: Conceptualization, Funding acquisition, Methodology, Resources, Supervision, Writing – review & editing.

## Supplementary material

Supplementary Figure 1 A representative experiment showing inhibition of *B. divergens* growth with application of Yoda1 or Go6983, displayed with non-linear regression curve fit.

Supplementary Figure 2
**(A)** Dose-response of *P. falciparum* 3D7 growth for the indicated compounds identified in this study to exhibit antiparasitic activity, displayed with non-linear regression curve fit. **(B)** The IC50s of chloroquine (top) or mefloquine (bottom) in the absence or presence of fixed doses of the indicated compounds. *P* values are shown for comparisons for which Student’s t test indicate a significant difference (<0.05).

Supplementary Figure 3 Hemolysis at 48 and 72h plotted in relation to phosphatidylserine exposure signal at 24h. The slopes for linear regression analyses are significantly non-zero (*P*<0.0001), with R^2^ values of 0.60 (48h data) and 0.78 (72h data). The boxed component of the plot in the left panel is rescaled and represented in the right panel. Source data are from **Supplementary Table 1**.

Supplementary Table 1 Compound-induced hemolysis and phosphatidylserine exposure.

Supplementary Table 2 Activity of compounds in RBCs and other mammalian cells.

## References

[B1] Adderley J. D. John von Freyend S. Jackson S. A. Bird M. J. Burns A. L. Anar B.. (2020). Analysis of erythrocyte signalling pathways during Plasmodium falciparum infection identifies targets for host-directed antimalarial intervention. Nat. Commun. 11, 4015. doi: 10.1038/s41467-020-17829-7 32782246 PMC7419518

[B2] Arang N. Kain H. S. Glennon E. K. Bello T. Dudgeon D. R. Walter E. N. F.. (2017). Identifying host regulators and inhibitors of liver stage malaria infection using kinase activity profiles. Nat. Commun. 8, 1232. doi: 10.1038/s41467-017-01345-2 29089541 PMC5663700

[B3] Asada M. Goto Y. Yahata K. Yokoyama N. Kawai S. Inoue N.. (2012). Gliding motility of babesia bovis merozoites visualized by time-lapse video microscopy. PloS One 7, e35227. doi: 10.1371/journal.pone.0035227 22506073 PMC3323635

[B4] Boulet C. Doerig C. D. Carvalho T. G. (2018). Manipulating eryptosis of human red blood cells: A novel antimalarial strategy? Front. Cell. Infection Microbiol. 8. doi: 10.3389/fcimb.2018.00419 PMC6284368 30560094

[B5] Brizuela M. Huang H. M. Smith C. Burgio G. Foote S. J. McMorran B. J. (2014). Treatment of erythrocytes with the 2-cys peroxiredoxin inhibitor, conoidin A, prevents the growth of plasmodium falciparum and enhances parasite sensitivity to chloroquine. PloS One 9, e92411. doi: 10.1371/journal.pone.0092411 24699133 PMC3974718

[B6] Cahalan S. M. Lukacs V. Ranade S. S. Chien S. Bandell M. Patapoutian A. (2015). Piezo1 links mechanical forces to red blood cell volume. eLife 4, e07370. doi: 10.7554/eLife.07370.013 26001274 PMC4456639

[B7] Chandramohanadas R. Davis P. H. Beiting D. P. Harbut M. B. Darling C. Velmourougane G.. (2009). Apicomplexan parasites co-opt host calpains to facilitate their escape from infected cells. Science 324, 794–797. doi: 10.1126/science.1171085 19342550 PMC3391539

[B8] Chien D. H. Pantaleo A. Keseley K. R. Noomuna P. Putt K. S.. (2021). Imatinib Augments Standard Malaria Combination Therapy without Added Toxicity. The Journal of Experimental Medicine 218, e20210724. doi: 10.1084/jem.20210724 34436509 PMC8404470

[B9] Deavall D. G. Martin E. A. Horner J. M. Roberts R. (2012). Drug-induced oxidative stress and toxicity. J. Toxicol. 2012, e645460. doi: 10.1155/2012/645460 PMC3420138 22919381

[B10] Dreischer P. Duszenko M. Stein J. Wieder T. (2022). Eryptosis: programmed death of nucleus-free, iron-filled blood cells. Cells 11, 503. doi: 10.3390/cells11030503 35159312 PMC8834305

[B11] Dziekan J. M. Yu H. Chen D. Dai L. Wirjanata G. Larsson A.. (2019). Identifying purine nucleoside phosphorylase as the target of quinine using cellular thermal shift assay. Sci. Trans. Med. 11, eaau3174. doi: 10.1126/scitranslmed.aau3174 30602534

[B12] Eberhard M. Ferlinz K. Alizzi K. Cacciato P. M. Faggio C. Föller M.. (2010). FTY720-induced suicidal erythrocyte death. Cell Physiol. Biochem. 26, 761–766. doi: 10.1159/000322343 21063113

[B13] Elsworth B. Keroack C. Rezvani Y. Paul A. Barazorda K. Tennessen J.. (2023). Babesia divergens egress from host cells is orchestrated by essential and druggable kinases and proteases. Res. Sq, rs.3.rs–2553721. doi: 10.21203/rs.3.rs-2553721/v1 PMC12872469 41593365

[B14] Grüring C. Moon R. W. Lim C. Holder A. A. Blackman M. J. Duraisingh M. T. (2014). Human red blood cell-adapted Plasmodium knowlesi parasites: a new model system for malaria research. Cell. Microbiol. 16, 612–620. doi: 10.1111/cmi.12275 24506567 PMC4004062

[B15] Hanna J. C. Corpas-Lopez V. Seizova S. Colon B. L. Bacchetti R. Hall G. M. J.. (2023). Mode of action studies confirm on-target engagement of lysyl-tRNA synthetase inhibitor and lead to new selection marker for Cryptosporidium. Front. Cell. Infection Microbiol. 13. doi: 10.3389/fcimb.2023.1236814 PMC10436570 37600947

[B16] Isberner N. Gesierich A. Balakirouchenane D. Schilling B. Aghai-Trommeschlaeger F. Zimmermann S.. (2022). Monitoring of dabrafenib and trametinib in serum and self-sampled capillary blood in patients with BRAFV600-mutant melanoma. Cancers (Basel) 14, 4566. doi: 10.3390/cancers14194566 36230489 PMC9558510

[B17] Jezewski A. J. Lin Y.-H. Reisz J. A. Culp-Hill R. Barekatain Y. Yan V. C.. (2021). Targeting host glycolysis as a strategy for antimalarial development. Front. Cell. Infection Microbiol. 11. doi: 10.3389/fcimb.2021.730413 PMC8482815 34604112

[B18] Kaufmann S. H. E. Dorhoi A. Hotchkiss R. S. Bartenschlager R. (2018). Host-directed therapies for bacterial and viral infections. Nat. Rev. Drug Discovery 17, 35–56. doi: 10.1038/nrd.2017.162 28935918 PMC7097079

[B19] Kuypers F. A. Lewis R. A. Hua M. Schott M. A. Discher D. Ernst J. D.. (1996). Detection of altered membrane phospholipid asymmetry in subpopulations of human red blood cells using fluorescently labeled annexin V. Blood 87, 1179–1187. doi: 10.1182/blood.V87.3.1179.bloodjournal8731179 8562945

[B20] Lambros C. Vanderberg J. P. (1979). Synchronization of plasmodium falciparum erythrocytic stages in culture. J. Parasitol. 65, 418–420. doi: 10.2307/3280287 383936

[B21] Lim C. Hansen E. DeSimone T. M. Moreno Y. Junker K. Bei A.. (2013). Expansion of host cellular niche can drive adaptation of a zoonotic malaria parasite to humans. Nat. Commun. 4, 1638. doi: 10.1038/ncomms2612 23535659 PMC3762474

[B22] Lobo C. A. Cursino-Santos J. R. Singh M. Rodriguez M. (2019). Babesia divergens: A drive to survive. Pathogens 8, 95. doi: 10.3390/pathogens8030095 31269710 PMC6789513

[B23] Lohia R. Allegrini B. Berry L. Guizouarn H. Cerdan R. Abkarian M.. (2023). Pharmacological activation of PIEZO1 in human red blood cells prevents Plasmodium falciparum invasion. Cell. Mol. Life Sci. 80, 124. doi: 10.1007/s00018-023-04773-0 37071200 PMC10113305

[B24] Lu K.-Y. Quan B. Sylvester K. Srivastava T. Fitzgerald M. C. Derbyshire E. R. (2020). Plasmodium chaperonin TRiC/CCT identified as a target of the antihistamine clemastine using parallel chemoproteomic strategy. Proc. Natl. Acad. Sci. U.S.A. 117, 5810–5817. doi: 10.1073/pnas.1913525117 32127489 PMC7084109

[B25] Lupescu A. Shaik N. Jilani K. Zelenak C. Lang E. Pasham V.. (2012). Enhanced Erythrocyte Membrane Exposure of Phosphatidylserine Following Sorafenib Treatment: An *in vivo* and *in vitro* Study. Cell Physiol. Biochem. 30, 876–888. doi: 10.1159/000341465 22907570

[B26] Mandt R. E. K. Lafuente-Monasterio M. J. Sakata-Kato T. Luth M. R. Segura D. Pablos-Tanarro A.. (2019). *In vitro* selection predicts malaria parasite resistance to dihydroorotate dehydrogenase inhibitors in a mouse infection model. Sci. Trans. Med. 11, eaav1636. doi: 10.1126/scitranslmed.aav1636 PMC7444640 31801884

[B27] McClure N. S. Day T. (2014). A theoretical examination of the relative importance of evolution management and drug development for managing resistance. Proc. Biol. Sci. 281, 20141861. doi: 10.1098/rspb.2014.1861 25377456 PMC4240990

[B28] Menard D. Dondorp A. (2017). Antimalarial drug resistance: A threat to malaria elimination. Cold Spring Harb. Perspect. Med. 7, a025619. doi: 10.1101/cshperspect.a025619 28289248 PMC5495053

[B29] Millholland M. G. Mishra S. Dupont C. D. Love M. S. Patel B. Shilling D.. (2013). A host GPCR signaling network required for the cytolysis of infected cells facilitates release of apicomplexan parasites. Cell Host Microbe 13, 15–28. doi: 10.1016/j.chom.2012.12.001 23332153 PMC3638031

[B30] Paoletta M. S. Laughery J. M. Arias L. S. L. Ortiz J. M. J. Montenegro V. N. Petrigh R.. (2021). The key to egress? Babesia bovis perforin-like protein 1 (PLP1) with hemolytic capacity is required for blood stage replication and is involved in the exit of the parasite from the host cell. Int. J. Parasitol. 51, 643–658. doi: 10.1016/j.ijpara.2020.12.010 33753093

[B31] Paul A. S. Moreira C. K. Elsworth B. Allred D. R. Duraisingh M. T. (2016). Extensive shared chemosensitivity between malaria and babesiosis blood-stage parasites. Antimicrobial Agents Chemotherapy 60, 5059–5063. doi: 10.1128/AAC.00928-16 27246780 PMC4958166

[B32] Prudencio M. Mota M. M. (2013). Targeting host factors to circumvent anti-malarial drug resistance. Curr. Pharm. Design 19, 290–299. doi: 10.2174/138161213804070276 22973886

[B33] Rezvani Y. Keroack C. D. Elsworth B. Arriojas A. Gubbels M.-J. Duraisingh M. T.. (2022). Comparative single-cell transcriptional atlases of Babesia species reveal conserved and species-specific expression profiles. PloS Biol. 20, e3001816. doi: 10.1371/journal.pbio.3001816 36137068 PMC9531838

[B34] Rizk M. A. El-Sayed S.A.E.-S. Alkhoudary M. S. Alsharif K. F. Abdel-Daim M. M. Igarashi I. (2021). Compounds from the medicines for malaria venture box inhibit *in vitro* growth of babesia divergens, a blood-borne parasite of veterinary and zoonotic importance. Molecules 26, 7118. doi: 10.3390/molecules26237118 34885700 PMC8658764

[B35] Rizk M. A. El-Sayed S.A.E.-S. El-Khodery S. Yokoyama N. Igarashi I. (2019). Discovering the in *vitro* potent inhibitors against Babesia and Theileria parasites by repurposing the Malaria Box: A review. Veterinary Parasitol. 274, 108895. doi: 10.1016/j.vetpar.2019.07.003 31494399

[B36] Scovino A. M. Totino P. R. R. Morrot A. (2022). Eryptosis as a new insight in malaria pathogenesis. Front. Immunol. 13. doi: 10.3389/fimmu.2022.855795 PMC9136947 35634341

[B37] Scully E. J. Shabani E. Rangel G. W. Grüring C. Kanjee U. Clark M. A.. (2019). Generation of an immortalized erythroid progenitor cell line from peripheral blood: A model system for the functional analysis of Plasmodium spp. invasion. Am. J. Hematol. 94, 963–974. doi: 10.1002/ajh.25543 31148215 PMC6984401

[B38] Sicard A. Semblat J.-P. Doerig C. Hamelin R. Moniatte M. Dorin-Semblat D.. (2011). Activation of a PAK-MEK signalling pathway in malaria parasite-infected erythrocytes. Cell Microbiol. 13, 836–845. doi: 10.1111/cmi.2011.13.issue-6 21371233 PMC3123749

[B39] Sisquella X. Nebl T. Thompson J. K. Whitehead L. Malpede B. M. Salinas N. D.. (2017). Plasmodium falciparum ligand binding to erythrocytes induce alterations in deformability essential for invasion. eLife 6, e21083. doi: 10.7554/eLife.21083.015 28226242 PMC5333951

[B40] van Schalkwyk D. A. Moon R. W. Blasco B. Sutherland C. J. (2017). Comparison of the susceptibility of Plasmodium knowlesi and Plasmodium falciparum to antimalarial agents. J. Antimicrobial Chemotherapy 72, 3051–3058. doi: 10.1093/jac/dkx279 PMC5890772 28961865

[B41] Voorhis W. C. V. Adams J. H. Adelfio R. Ahyong V. Akabas M. H. Alano P.. (2016). Open source drug discovery with the malaria box compound collection for neglected diseases and beyond. PloS Pathog. 12, e1005763. doi: 10.1371/journal.ppat.1005763 27467575 PMC4965013

[B42] Wadud R. Hannemann A. Rees D. C. Brewin J. N. Gibson J. S. (2020). Yoda1 and phosphatidylserine exposure in red cells from patients with sickle cell anaemia. Sci. Rep. 10, 20110. doi: 10.1038/s41598-020-76979-2 33208899 PMC7674503

[B43] Waked R. Krause P. J. (2022). Human babesiosis. Infect. Dis. Clinics North America Lyme Dis. Expanded Spectr. Blacklegged Tick-Borne Infections 36, 655–670. doi: 10.1016/j.idc.2022.02.009 36116841

[B44] Wei L. Adderley J. Leroy D. Drewry D. H. Wilson D. W. Kaushansky A.. (2021). Host-directed therapy, an untapped opportunity for antimalarial intervention. Cell Rep. Med. 2, 100423. doi: 10.1016/j.xcrm.2021.100423 34693368 PMC8524702

[B45] Wesseling M. C. Wagner-Britz L. Huppert H. Hanf B. Hertz L. Nguyen D. B.. (2016). Phosphatidylserine exposure in human red blood cells depending on cell age. CPB 38, 1376–1390. doi: 10.1159/000443081 27007671

[B46] Wicht K. J. Mok S. Fidock D. A. (2020). Molecular mechanisms of drug resistance in plasmodium falciparum malaria. Annu. Rev. Microbiol. 74, 431–454. doi: 10.1146/annurev-micro-020518-115546 32905757 PMC8130186

[B47] Wu X. Dayanand K. K. Thylur R. P. Norbury C. C. Gowda D. C. (2017). Small molecule–based inhibition of MEK1/2 proteins dampens inflammatory responses to malaria, reduces parasite load, and mitigates pathogenic outcomes. J. Biol. Chem. 292, 13615–13634. doi: 10.1074/jbc.M116.770313 28679535 PMC5566520

[B48] Zelenak C. Föller M. Velic A. Krug K. Qadri S. M. Viollet B.. (2011). Proteome analysis of erythrocytes lacking AMP-activated protein kinase reveals a role of PAK2 kinase in eryptosis. J. Proteome Res. 10, 1690–1697. doi: 10.1021/pr101004j 21214270

