# Supplementary figures and images for: Inhibition of malaria and babesiosis parasites by putative red blood cell targeting small molecules
Source: Front Cell Infect Microbiol. 2024 Mar 20;14:1304839. doi: 10.3389/fcimb.2024.1304839 (PMC10988762; doi:10.3389/fcimb.2024.1304839)

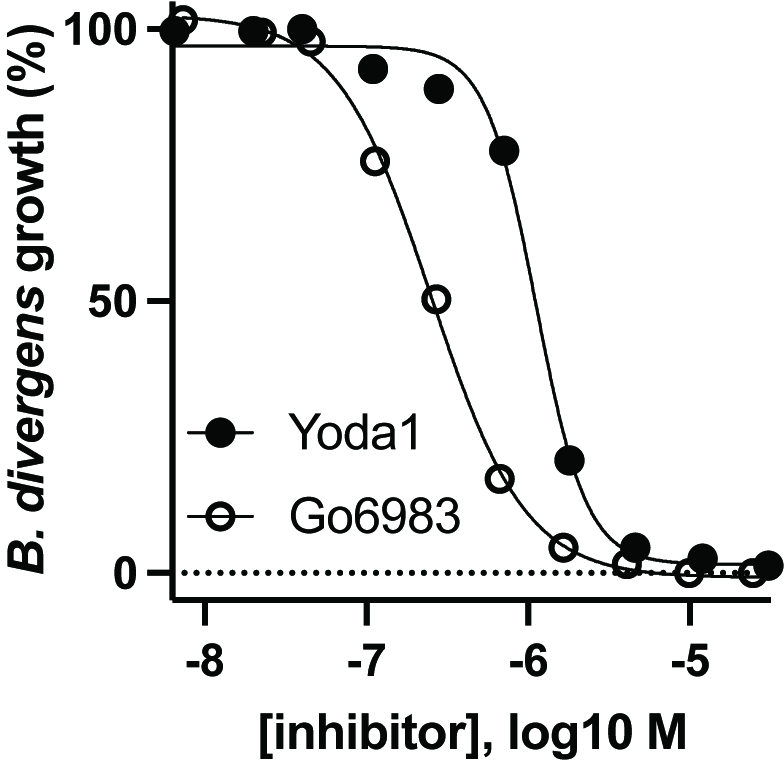

Supplement: Supplementary Figure 1 — A representative experiment showing inhibition of B. divergens growth with application of Yoda1 or Go6983, displayed with non-linear regression curve fit. [file Image_1.tif]

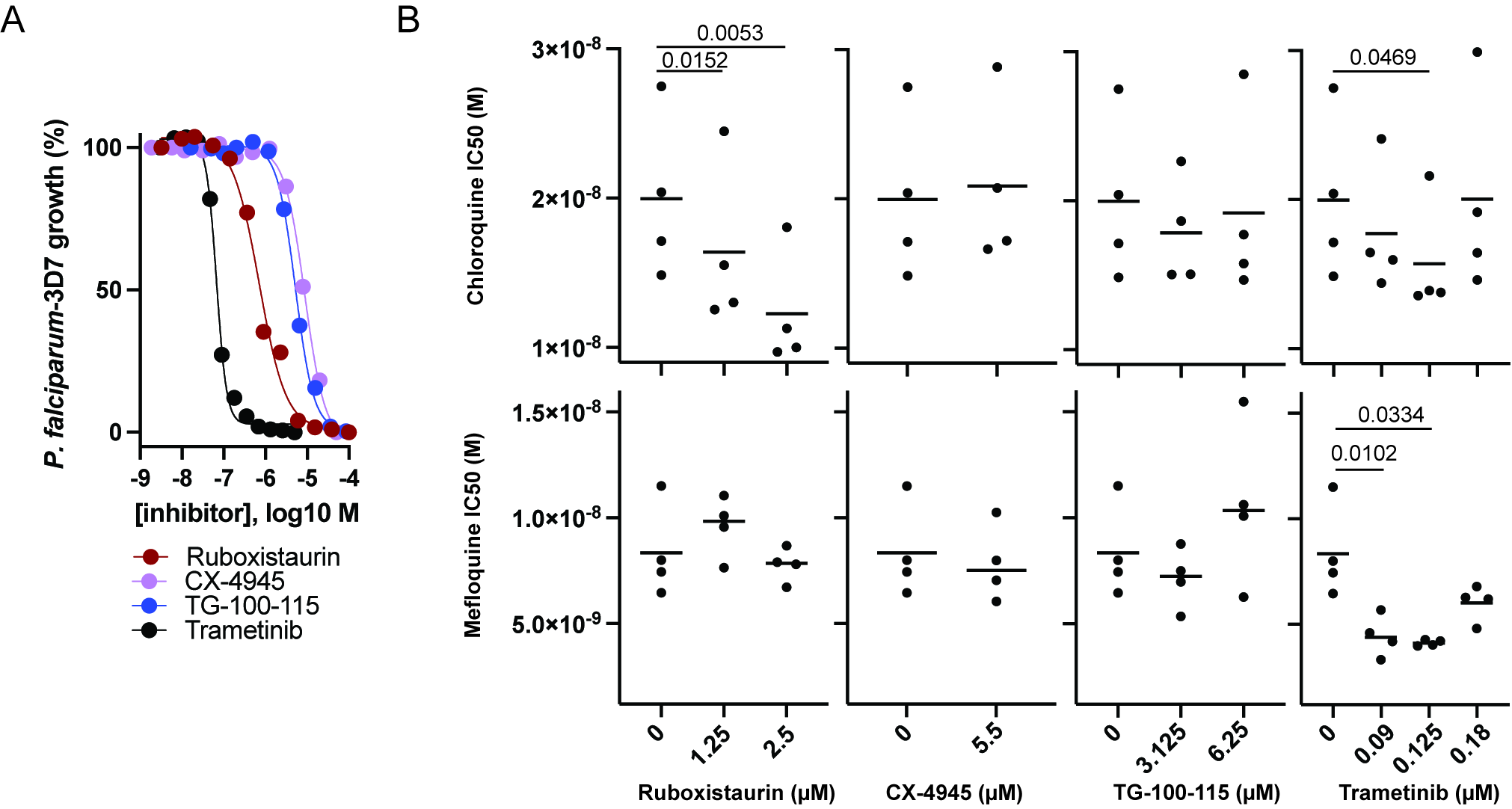

Supplement: Supplementary Figure 2 — (A) Dose-response of P. falciparum 3D7 growth for the indicated compounds identified in this study to exhibit antiparasitic activity, displayed with non-linear regression curve fit. (B) The IC50s of chloroquine (top) or mefloquine (bottom) in the absence or presence of fixed doses of the indicated compounds. P values are shown for comparisons for which Student’s t test indicate a significant difference (<0.05). [file Image_2.tif]

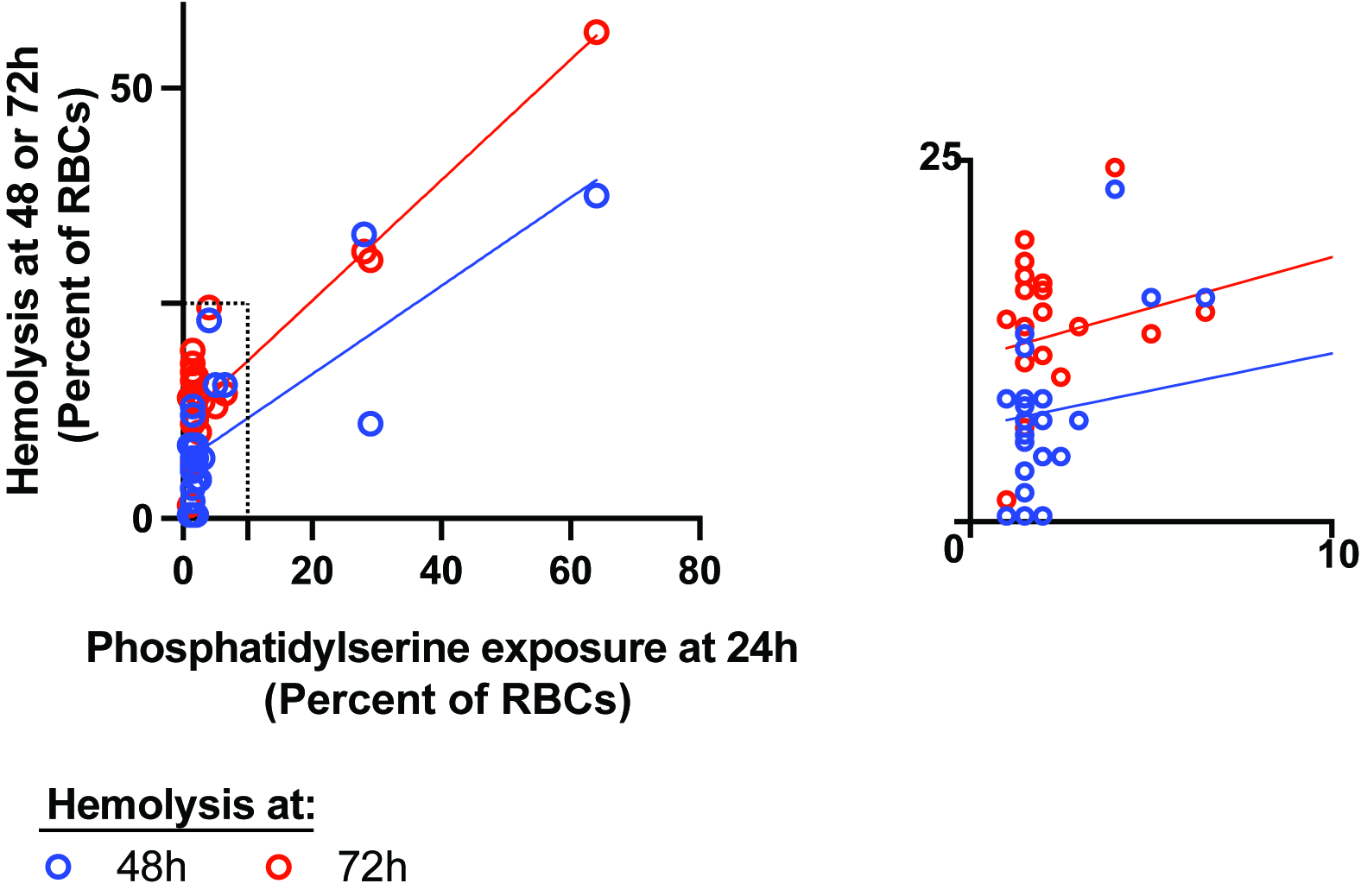

Supplement: Supplementary Figure 3 — Hemolysis at 48 and 72h plotted in relation to phosphatidylserine exposure signal at 24h. The slopes for linear regression analyses are significantly non-zero (P<0.0001), with R2 values of 0.60 (48h data) and 0.78 (72h data). The boxed component of the plot in the left panel is rescaled and represented in the right panel. Source data are from Supplementary Table 1 . [file Image_3.tif]
