# Supplementary material for: Inhibition of malaria and babesiosis parasites by putative red blood cell targeting small molecules
Source: Front Cell Infect Microbiol. 2024 Mar 20;14:1304839. doi: 10.3389/fcimb.2024.1304839 (PMC10988762; doi:10.3389/fcimb.2024.1304839)
Supplement: Supplementary Table 1 — Compound-induced hemolysis and phosphatidylserine exposure. [file Table_1.docx]

|  | ***Hemolysis (%)**** | |  | ***Phosphatidylserine exposure (%)**** | | |
| --- | --- | --- | --- | --- | --- | --- |
|  | ***24h*** | ***48h*** | ***72h*** | ***24h*** | ***48h*** | ***72h*** |
| *Trametinib* | *-1* | *7* | *15* | *2* | *2* | *5* |
| *Calpeptin* | *0* | *8* | *17* | *2* | *2* | *5* |
| *Crizotinib* | *1* | *9* | *14* | *1* | *2* | *5* |
| *AA74-1* | *-6* | *-1* | *2* | *1* | *2* | *5* |
| *Bafetinib* | *1* | *6* | *4* | *2* | *2* | *4* |
| *U-73122* | *-3* | *16* | *13* | *5* | *9* | *11* |
| *FTY720* | *4* | *23* | *25* | *4* | *21* | *34* |
| *Go6983* | *-7* | *5* | *12* | *2* | *2* | *4* |
| *SB590885* | *-4* | *4* | *11* | *2* | *2* | *4* |
| *U0126* | *3* | *9* | *20* | *2* | *4* | *9* |
| *IPA-3* | *-14* | *11* | *30* | *29* | *35* | *35* |
| *TG-100-115* | *-1* | *12* | *18* | *2* | *3* | *5* |
| *Sotrastaurin* | *-7* | *9* | *17* | *2* | *2* | *4* |
| *Auphen* | *-7* | *5* | *10.* | *3* | *10.* | *24* |
| *Yoda1* | *7* | *38* | *57* | *64* | *100.* | *100.* |
| *Imatinib* | *-2* | *2* | *7* | *2* | *2* | *4* |
| *Ruboxistaurin* | *-1* | *6* | *14* | *2* | *2* | *6* |
| *Calmidazolium* | *-6* | *7* | *14* | *3* | *4* | *9* |
| *LY3214996* | *-1* | *13* | *16* | *2* | *2* | *5* |
| *Sorafenib* | *-6* | *16* | *15* | *7* | *12* | *21* |
| *CX-4945* | *-4* | *-1* | *16* | *2* | *2* | *3* |
| *KN-93* | *-4* | *7* | *16* | *2* | *2* | *4* |

*mean of 2 experiments.
