# Supplementary material for: Inhibition of malaria and babesiosis parasites by putative red blood cell targeting small molecules
Source: Front Cell Infect Microbiol. 2024 Mar 20;14:1304839. doi: 10.3389/fcimb.2024.1304839 (PMC10988762; doi:10.3389/fcimb.2024.1304839)
Supplement: Supplementary Table 2 — Activity of compounds in RBCs and other mammalian cells. [file Table_2.docx]

| ***Compound*** | ***Pf3D7-IC50 (µM)*** | ***Micromolar IC50 (cell type)**** | ***RBC assay*** | ***PMID*** |
| --- | --- | --- | --- | --- |
| *Trametinib* | *0.09* | *0.0016-0.0048 (MEF)* |  | *29336889* |
| *Calpeptin* | *1* | *74.2 (SW1990)* |  | *27487486* |
| *Crizotinib* | *0.7* | *0.311 (H2228)* |  | *30333870* |
| *AA74-1* | *0.25* | *nd* |  |  |
| *Bafetinib* | *2* | *0.0011 (K562); 0.0022 (293T)* |  | *19662183* |
| *U-73122* | *4* | *nd* |  |  |
| *FTY720* | *3.9* | *5-10 (RBC)* | *PS exposure* | *21063113* |
| *Go6983* | *2* | *0.1 (AP-1 transcription factor activation)* |  | *17175522* |
| *SB590885* | *0.7* | *1.1-6.1 (human epithelial cells)* |  | *17145850* |
| *U0126* | *5.3* | *19.4 (anchorage formation, HCT-116); 0.293 (ELK1-luciferase assay, HeLa)* | *Inhibits anchorage formation in HCT-116 cells* | *15225706* |
| *IPA-3* | *5* | *<5 (RBC)* | *PS exposure* | *21214270* |
| *TG-100-115* | *9* | *nd* |  |  |
| *Sotrastaurin* | *5* | *0.090 (alloresponsive T cell proliferation)* |  | *24131367* |
| *Auphen* | *1.1* | *0.8 (RBC)* | *AQP3 glycerol conductance* | *22624030* |
| *Yoda1* | *4.2* | *~2 (RBC)* | *PS exposure in sickle RBCs* | *33208899* |
| *Imatinib* | *3.3* | *<5 (RBC)* | *Band 3 phosphorylation* | *27768734* |
| *Ruboxistaurin* | *0.9* | *nd* |  |  |
| *Calmidazolium* | *0.5* | *nd* |  |  |
| *LY3214996* | *5.2* | *nd* |  |  |
| *Sorafenib* | *4.9* | *>10 (RBC)* | *PS exposure* | *22907570* |
| *CX-4945* | *9* | *1.7-20 (breast cancer cell line panel)* |  | *21159648* |
| *KN-93* | *3.9* | *6-12 (serum- or growth factor induced fibroblasts)* |  | *8519682* |

* For non-RBC cell types, assay is cellular proliferation unless otherwise noted
